# Book Reviews

**Published:** 1816-06

**Authors:** 


### CRITICAL ANALYSIS
### *OF RECENT PUBLICATIONS.*

*An Experimental Inquiry into the Nature, Cause, and Varieties of the Arterial Pulse; and into certain other properties of the larger Arteries in Animals, with Warm Blood; illustrated by Engravings;* by CALEB HILLIER PARRY, M.D. F.R.S. Member of the College of Physicians, London; &c. &c.—Underwood, 1816.

IT was a notion which formed a part of ancient physiology, that the arteries were a system of channels, destined to convey animal spirits to the different parts of the body. Modern physiology may boast an advantage over the ancient, inasmuch as it has been fully ascertained, that, although the arteries may not be without their spirits, they certainly do convey blood. It is not for want of labour or ingenuity, for both have been lavished on the subject, that we are almost under the necessity of contenting ourselves with this gentle boast; but it is, we apprehend, owing to some certain difficulties which are common in matters of inference, that this labour and this ingenuity have been attended with so few other unequivocal results.

In points where absolute certainty is unattainable, it is perhaps better to entertain a positive belief, although that belief may be erroneous, than, unsatisfied, to be perpetually fermenting our wits, in order to strengthen our conviction. So far the ancients had the advantage of us; for, with them, the leapings of the arteries were a sort of pastime and recreation, creation, in which their spirits were prone to indulge; but we, suffering under the curse of a little knowledge, have discovered that these same leapings may be as well attributed to the blood, as to the spirits, of the arteries; and the ardour of research has served but to multiply the intricacies which it aspired to obviate.

It was thought by the discoverer of the circulation, that the whole mass of blood was propelled through its several channels by the force which the heart alone exerted in the motion which is termed its systole. Copious and learned were the disquisitions which followed the promulgation of this discovery. Spiritualists, mechanists, chemists, and even poets, have said or sung upon this theme, each of them in their own way. At length the alternatives were simplified; and the labours of our fore-fathers have left us now to decide, whether the circulation is performed by the mere action of the heart; or whether the arteries, imitating the action of the heart, contribute by their contractions and dilatations, to accomplish the same end.

Since the time of Haller, it has been commonly believed that arteries possessed an independent power of action; the faculties of contraction and dilatation were thought to be distinguished by every person who could feel a pulse. The possession of these faculties came, however, in time to be denied; and the motions of the arteries were said to consist only of a change of place. It was in this state of the question, when each of the above alternatives had its advocates; when some believed that an artery contributed towards the circulation, by a contraction and dilatation of its parietes; others asserting, at least, that an artery was capable of contraction and dilatation; and others agreeing in the opinion, that an artery had no other than a loco-motion; this was the state of the question, when the further elucidation of it was undertaken by the respectable author of the work before us.

Dr. Parry relates a series of experiments, which, as they are very numerous, our limits will not permit us to follow in detail. It must suffice, therefore, for the present, to give the following general description. The experiments were made upon sheep, rabbits, and horses. They consisted commonly in denuding one or both carotids, an aorta, or a femoral artery; and in observing their motions under different circumstances, as under the action of one ligature, or of two ligatures, with the naked eye, with the aid of a magnifier, &c. The result of this examination is, that in no instance, by the minutest scrutiny, could any pulsation, consisting of contraction and dilatation, be discerned. It was seen that an artery was not perfectly quiescent, but that its movements were those only of elongation and recovery.

It is attempted also to illustrate copiously, by experiment, the varieties of arterial contraction; to assign in what cases the contraction of an artery is attributable to its tonic power; and in what to its elasticity. It is also attempted to estimate the comparative force of these two causes of contraction. The experiments are given in a successive detail, uninterrupted by comment. To this detail is subjoined, a discussion of the topics to which the experiments relate; which discussion is founded principally upon these experiments, with occasional comparisons between the author's own results and those of preceding writers, more especially of Mr. Hunter, who laboured ingeniously in the same field. The experiments appear to have been conducted with accuracy, their results to have been correctly observed, and to be reported faithfully.

The following scale, illustrative of the effects of blood-letting upon the caliber and action of arteries, though perhaps too distantly and ambiguously related with practice to have any great weight in its decisions, may be extracted for the consideration of our readers; the subject was a small ewe-sheep.—P. 46.

| | *Circumf. of the Artery.* | *Pulse.* | *Respiration.* |
|---|---|---|---|
| | Inch. | | |
| Before bleeding - - | $\frac{222}{400}$ | 124, and regular | 24, chiefly by the diaphragm |
| Eight oz. of blood taken from the left jugular - | $\frac{196}{400}$ | 72 - - - | 24 ditto |
| 9 ounces taken away - - | $\frac{178}{400}$ | 68 - - - | 24, and equal |
| 8 ounces - - - - - | $\frac{170}{400}$ | 80, and weaker - | 20, and unequal |
| 8 ounces - - - - | $\frac{145}{400}$ | 100, more jerking, and apparently stronger | 14, and irregular in every respect |
| 8 ounces - - - - | $\frac{127}{400}$ | 116, still more bounding - - | 24, respiration quicker and harder |
| 8 ounces - - - - (sheep faint) | $\frac{140}{400}$ | 116, as before - | 20, irregular as to frequency and depth, and a good deal by the thorax |
| 8 ounces, flowing freely at the beginning, but gradually more slowly - | $\frac{141}{400}$ | 168, very bounding, and tolerably strong - - | 16, irregular; the elevation of the thorax being often alternate with the descent of the diaphragm |
| 2 ounces - - - - (the jaw of the animal fell) | $\frac{145}{400}$ | none - - | gone. |
| 18 minutes after death - | $\frac{158}{400}$ | | |
| 27 ditto - - - - | $\frac{160}{400}$ | | |
| 33 ditto - - - | $\frac{169}{400}$ | | |

We

We pass over the section on the structure of arteries, to which succeeds one on the powers of arteries, referring principally to their contraction before mentioned. The want of uniformity in the results of an inquiry, designed to ascertain the comparative force of the elastic and tonic powers of arteries, is thus acknowledged:—"It is, indeed, probable, that the proportion between the tonic and elastic powers may greatly vary, not only in different arteries, and in different animals, but in the same individual animal at different times. So also the distention of an artery by its contained blood, may vary with the same modifications as those just expressed; so that any single experiment, such as I have described, can only be considered as relative to those circumstances. In the mean while, the general principle of the existence of two moving powers in arteries of warm-blooded animals is, by these experiments, illustrated;"* and, we might add, illustrated in no mean degree. The conclusions of the author on this subject are different from those of Mr. Hunter; for the particulars we must refer the reader to the work itself.

Dr. Parry explains the fact, that the arteries, after death, are usually found empty, by supposing that "these vessels were contracted, by their tonicity, beyond the degree which would otherwise have been permitted by their elasticity; but the tonicity soon ceasing, with the other powers of organic life, the arteries would be dilated by their elasticity, and would therefore remain more or less robbed of their blood." It is attempted to confirm this explanation by the following experiment:—"The carotid of a living ram, measured $\frac{260}{400}$th of an inch; the artery, at the same spot, five minutes after apparent death, measured only $\frac{190}{400}$th of an inch; thus the circumference appeared to have been reduced $\frac{70}{400}$th of an inch." This degree of contraction we conceive not to be sufficient for the effect imputed to it. We have sometimes remarked, that a portion of an artery in the dead subject has been totally destitute of blood, and in general the quantity contained is exceedingly small; so that in the former case, if the blood is evacuated from an artery by compression, it would require a temporary obliteration; in the latter, a degree of contraction (correspondent with the blood found in it) of at least three-fourths of its natural caliber. The fact in question is more readily explained by the doctrine which supposes the circulation to be in part maintained by the action of the arteries, than that which is now intended to supersede it. According to the former, the blood would be expelled from the arteries by that last exer-

* Page 68.

tion of the faculty of contraction, which is synchronous with the termination of life.

The tonic and elastic powers of arteries are opposed to each other in their respective actions, and both are liable to be overcome by the distending force of the blood. An artery unduly contracted by the tonic power, this ceasing, recovers its caliber by its elasticity, as after death ; and the momentum of the blood prevailing, the artery is said to be kept in a forced state, in which the tonic power which would contract, and the elastic which would preserve the uniformity of its area, are inadequate to oppose the cause of distention. The author limits the power of the middle coat of arteries to that of contraction, affirming that there is no evidence of a power of elongation by which these vessels might be dilated, except by their elasticity, and by the momentum of the blood.* We cannot entirely acquiesce in this conclusion, since it appears irreconcilable with some of the most conspicuous and generally acknowledged phenomena of inflammation ; we allude chiefly to that perceptible enlargement of the arteries, which takes place in this process. The inflammation may be excited in the finger by the puncture of a needle, or by any foreign substance lodged under the nail. We would ask, what relation is there between such a cause of injury and the *elastic* power of arteries, which is the only one assigned by Dr. P., except the distending one of the blood, capable, under any circumstances, of increasing their caliber? We are assured that such foreign substance has no relation with the elasticity of an artery, this being a property of dead matter, and the same cause being attended in the dead subject with no such effect. Nor can we imagine that the foreign substance has any direct relation with the blood, by which a greater quantity than natural of this fluid is poured into the digital arteries, thereby distending them ; this cannot be imagined, because the blood is passive. The cause of dilatation must be looked for then in the arteries themselves ; and if it happens neither by their tonic power, nor by their elastic power, a third must be inferred, which, in agreement with the perceptible phenomena, might be called one of dilatation. That a similar power alternates with one of contraction in the heart itself, we are inclined to believe, as well from some observations of disease, as from the forcible dilatations of the ventricles which we have witnessed in a large animal, a few minutes after death, from loss of blood. But, as we are not aware of an accurate measurement having been made,

* Page 79, 80, &c.

the the design of which was to ascertain the comparative dilatation of the ventricles during the action of the heart, in an animal just deprived of its blood; and that degree, which, in the state of entire death, is preserved by the elastic powers of the heart,—we cannot, whilst the fact is thus undecided, affirm positively the conclusion which must be founded upon it.

The fourth section, and not the least interesting, treats "of the nature and cause of the arterial pulse." We cannot follow our author through the whole of this discussion; it must suffice, therefore, in a general way, to state the facts and conclusions maintained in this part of the subject. Dr. P. has frequently denuded the arteries of various animals, the result of which has been, that these vessels have appeared uniformly nearly in a state of rest. An artery, upon exposure, may be seen to be elongated and to recover its place; but in no instance, it is stated, are an alternate contraction and dilatation to be perceived. This fact, which has been remarked by many preceding physiologists, and which we shall consider at some length, is the basis of the explanation of the pulse afterwards proposed.

The evidence in regard to the fact, is of the following kind:—If an artery contracted and dilated, these motions would be seen when the vessel is exposed to the eye. Then the fact opposed to this is, that the contractions and dilatations of an artery may be felt, although they cannot be seen. Here is the testimony of one sense opposed to that of another:—which are we to prefer? In the first, the vessel is deprived of its covering, and so far is in a preternatural condition: in the second, there is no removal, no disturbance; a sense of contraction and dilatation is perceived by the finger, while the vessel is covered by its skin; and is, in every respect, save that it may be slightly compressed, in a natural state. If it be objected that the sense of touch is not applicable in this case, because touch implies pressure, it may as fairly be said that the sense of vision is inapplicable, because it requires that the natural covering of the vessel should be removed. But the author denies the efficiency of a covering to the artery in giving the pulse; observing, that "no one ever felt a pulse the better because a thick garment, or a steel gauntlet, intervened between the artery and the finger."* There is, however, this trifling difference between the natural covering of a vessel and a steel gauntlet, viz. that through the one the pulse might be

* Page 100.

felt distinctly, while through the other it cannot be felt at all.

Dr. Parry cannot imagine how the interposition of integuments can contribute towards the occurrence of contractions and dilatations in an artery; and that therefore the test of vision is the only one to be relied on. We know, from a thousand familiar instances, that the vital properties of any structure are liable to be influenced by a mechanical or chemical relation, either with adjoining parts, or with external agents. We see how vital properties first, and then the secretions, are changed by the admission of air into cavities. We would ask how long an animal may survive a simple exposure, without injury or profuse loss of blood, of the spinal marrow, of the brain, the heart, or even of the abdominal viscera? In truth, the function of every part that is covered, will be more or less influenced by a removal of its covering. It may be difficult to define with precision, the nature and varieties of this relation; but that such a relation exists, is sufficient to diminish our reliance upon results, proposed as natural, which are seen to take place only under preternatural circumstances.

But, in a question on the actions of arteries, it is not difficult to particularize a mode in which these actions may be dependent upon their coverings, setting aside the force of the general relation just now suggested. When an artery is covered by skin, a certain degree of pressure is made upon it, the effect of which will be to prevent an over-stretching of its muscular fibres by the force of the circulation.* When this covering is removed, the dilatation of the artery being no longer resisted, the blood distends it beyond the scope of its contractile power; and hence the tube in this place is kept, by a permanent distention, nearly in a state of rest. Nor can this explanation of the want of pulse in a denuded artery, be easily refuted; because, for the purpose of measurement, or a testimony of the visible kind, the artery must be placed in the state which involves our objection, namely, in one when deprived of its covering. That this remark is not without foundation, seems evident both from its being supported by many analogies in muscular phenomena, as well as from the following order of the facts—an artery felt through the skin, contracts and dilates;

* Such an effect of pressure is, in some degree, exemplified by the removal of that of the atmosphere, as in cupping, &c.; and the continued bleeding which follows the bite of a leech, evinces for how considerable a time the contractile power of an artery may be suspended by an undue dilatation.

remove remove the skin, it no longer contracts and dilates; interpose, as was suggested by Mr. Hunter, another medium, as an equivalent for skin, (tending equally to resist undue dilatation,) and then the contractions and dilatations of the vessel will become evident to both senses. It is but just to exhibit the author's explanation of the arterial pulse, in his own words:—

"When, by the contraction of the left ventricle, the blood included in it is forcibly expelled into the aorta, all these columns receive the shock of propulsion at the same instant. But the velocity, during this systole, being greater than during the diastole, the momentum, and consequently the impulse, in every direction, is also greater in the systole. When, therefore, an artery is compressed with the fingers in the usual mode of feeling the pulse, the blood, in consequence of the systole rushing into the artery with an increase of momentum, gives a stronger impulse of dilatation to the fingers than from the less momentum, which exists during the diastole, and thus produces the phenomenon of the pulse."*

Upon comparison of the pulse of arteries, situated at different distances from the heart, as of the radial and the temporal, or the inguinal and the carotid, we have remarked that their dilatations and contractions are synchronous. Of the truth of the explanation of this circumstance, we are not perfectly satisfied. The blood may exist in the vessels in continuous columns; but, as the quantity thrown out from the left ventricle at each systole, must dilate the aorta and nearer vessels, sooner than those which are remote, the dilatation of the several arteries should be rather successive than simultaneous; by which we mean that some time must be lost in the propagation of an impulse by a jet of blood from the heart to the remotest arteries, through a multiplicity of channels, under every variety of flexure.

The author remarks, "the coats of the carotids are so firm, that, when either impelled against any soft substance, or simply moved out of their place, these arteries readily recede, suffering no reduction of diameter, and therefore giving no sensation of a pulse."† This observation is made on the denuded carotid; but here again the result is different under an experiment as nearly similar as possible, while the integument is entire. Thus, if the finger is placed half an inch on the outer side of the carotid, in a thin subject, and pressure made against this vessel in the direction towards the trachea, the pulsation of the artery will be felt distinctly; and the same thing happens if the pressure is directed from the tracheal side of the artery, towards the mastoid muscle.

---

* Page 111. † Page 112.

In

In both these cases the artery is displaced laterally, and not compressed between two resisting substances.

The author next proceeds to explain why a pulse, in some surgical operations, is not to be felt; which is, that, if an artery is compressed on one side, there being no resistance on the other, the tube will be only bent; and no reduction of caliber occurring, the pulse, which is said to depend upon such reduction, will not be distinguished. Hence, the identity of an arterial tube is at all times to be ascertained, either by placing some resisting substance beneath the artery, while the finger depresses it from above, or by compressing the artery between the finger and thumb. This remark deserves to be borne in mind, as one which might be eminently useful in an important branch of operative surgery. At the same time, we conceive the fact to prove only, that under this state of pressure, the undue dilatation of the artery before mentioned being prevented, a jet of blood, correspondent with the systole of the heart, is perceptible to the touch; while the fact neither proves the correctness of our author's explanation of the pulse, nor militates against that which we have opposed it. But, that an action of the artery itself takes place in the common instance of feeling a pulse, is also indicated by the sensation communicated to the finger, independent of any *à priori* reasoning. We feel that the artery, having struck the finger in its dilatation, immediately suffers a diminution, which does not arise from the external pressure. If an artery, in the case alluded to, suffered a reduction of its size from no other cause than the pressure of the finger, it should present to the touch a sense of constant distention, with a distinct impulse at each systole of the heart. Whereas, the artery is perceptible only at the moment of its dilatation, and then sinks so considerably, that the sense of its existence is lost until its dilatation is repeated.

If two fingers are laid on the radial artery of a thin subject, and rolled backwards and forwards, lightly, over the vessel, the sensation will be that of a tube uniformly distended; that is, the artery will not, by this mode of feeling it, appear to contract and dilate. But this mode of examination is not to be relied on; for, if a stronger degree of pressure is exerted, and the caliber of the artery half obliterated, still no sensation of a pulse will occur under a similar movement of the finger. This fact, militating alike against both theories, appears to shew only that such a mode of examination is not adapted for the purpose.

We confess, on the subject of the arterial pulse, we are not yet quite satisfied; we cannot, however, but bestow our warmest warmest approbation upon the scientific manner in which Dr. Parry has conducted his inquiry. Our limits prevent a further examination of this part of the subject; we should otherwise draw out in array many other circumstances belonging to the physiology of arteries, with copious illustrations from disease, in support of that doctrine which we have professed ourselves unwilling to relinquish. We therefore conclude this extended article, with a short account of the fifth section, "on a further power of arteries."

Two cases are given, illustrated by neat engravings, of a power of reproduction, which, we believe, has never before been discovered to belong to the arterial trunks. Both carotids of a ram were tied on the 27th of September, 1815 (we presume 1814). On the 7th of August, 1815, this ram was killed and injected. The portion of the left carotid, obliterated by the ligature, was supplied "by five new ramifications, uniting, in different points, the extremity of the inferior portion of the old artery, with the superior portion;" and in this way was the circulation restored to its former channel. Although scepticism may urge many objections to considering these branches as new productions, we are in candour compelled to admit the fact. We think it next to impossible, that all these branches should have been overlooked at the time of tying the carotid, if they had existed previously to this operation. The fact is further confirmed by a second case of the same description, though a less perfect specimen than the last. The right carotid of a ram was tied on the 12th of October, 1814—the animal was killed on the 7th of September, 1815. Something more than two inches in length, of the original trunk, had disappeared, and three small branches united the extremities of the old artery. These branches were pervious, but not of a sufficient size to admit coarse injection. This is certainly a very interesting discovery; and we think it not improbable, as the attention of anatomists will now be directed to the point, but a similar provision for the re-establishment of the circulation, may at some future time be detected in the human subject. Indeed we see nothing in the fact which should either create astonishment, or excite the ardour of opposition. A process of a similar kind, but vastly more complex, takes place beyond all doubt in every example of extensive regeneration; as in the filling up and cicatrization of large ulcers, &c. where the vessels in the new growth, however numerous their anastamoses, communicate with the old trunks, through which their blood is received from, and returned to, the heart. We shall, we trust, at at a future time, be gratified with additional examples of this important discovery.

The short, but pleasing duty, now remains, of recommending this work to the attention of our scientific readers. There are few, we apprehend, whose views of the subject it embraces, may not be improved by its perusal. Every page of it bears evidence of acute discernment, and of a superior talent for observation. We think, however, that the same results might have been obtained by fewer experiments; and, while we regret that the superstructure of medical science should at any time be built upon the blood and sufferings of animals, like ourselves endowed with the faculty of sensation, we earnestly wish that all those who engage in experimental inquiries of this nature, would recollect whether the facts they seek for, or equivalent ones, may not be acquired either by collateral evidence, or gleaned from the accumulated records of past experience.

[Contrary to our established custom, we have admitted the above without examination of the work itself. The source, however, from which we receive it is so respectable as to justify our confidence.—We shall, in a few numbers, endeavour to do justice to Dr. Parry's Elements of Pathology, which are at this time under review.]

---

*An Essay on the Prevention and Cure of Insanity; with Observations on the Rules for the Detection of Pretenders to Madness.* By George Nesse Hill, Medical Surgeon, and Surgeon to the Benevolent Institution for the Delivery of Poor Married Women in Chester. 8vo. pp. 446. Longman and Co. London.

This performance has hitherto somehow escaped us, but we need no apology for adverting to it so long after its publication. Not only the importance of the subject, the matured experience of the author, but the particular manner in which the public attention is at this time directed to this most unfortunate condition of the species, imperiously call upon us to add every source of information in our power. Mr. Hill's work evinces much reading, much industry, and, what is most to the purpose, we have every reason to believe, much faithfulness. There is, also, throughout, a great attempt at method; but in this we conceive the author has unhappily failed. We shall, however, pursue the plan by which he has arranged his labours, which, with occasional extracts, will prove the fairest means of submitting the merits of the whole to the judgment of our readers.

The introduction is principally directed to show the insufficiency sufficiency of what has hitherto been published on madness. In this the author is, as might be expected, very successful. The unfinished publication of Dr. Arnold, the gloomy views of Pinel, the inconclusive though well-written treatise of Mr. Haslam, and several other writers, are glanced at with much candour, mixed with a sufficient air of sarcasm. The concluding part furnishes at once an insight into the author's intentions, and a foretaste of a verbosity which we confess became somewhat irksome before we had completed our task as reviewers.

"The reader," says Mr. Hill, "is particularly intreated to observe, that the two following axioms contain the principles which form the basis of the essay:

"First. That insanity is always a symptomatic disease.

"Secondly. That it is never a purely mental disease.

"'There are some truths,' says the late learned Bishop of Cloyne, 'so near and obvious to the mind, that a man need only open his eyes to see them.' The foregoing axioms are two of this description: how far their justness of assumption will be supported by incontestible evidence, remains to be seen, during the investigation of the subject; for the present they must be admitted as probable truths—ultimate conviction may be obtained by faithfully subjecting the plan hereafter proposed to the test of experience, that *dernier* court from whence there is no appeal. At the same time it must not be forgotten, that the grand aim is an endeavour to illustrate practical truths that rely principally upon experience; and that 'it is not a thing so easy as is conceived to convey the conceit of one man's mind into the mind of another, without loss or mistaking, especially in notions new and differing from those that are received.' *Bacon*, v. i. p. 392.

"Whilst detailing my opinions and practice, with considerable hesitation, to the world, I have presumed to appreciate the generous indulgence of all who will give attention to the subject proportionate to its desert; thus will they meet a fair examination, finally to receive approval or condemnation, from the most impartial tribunal.

"The scope of this essay will be learnt from what has been already advanced, and the following deductions, which the success of the mode of treatment recommended, have fully established, viz.

I. That Insanity — has always corporeal disease for its foundation.

II. ——— consists of but one species under two forms, viz. the Sthenic and Asthenic, or *Mania* and *Melancholia*.

III. ——— is not a hereditary disease in the vulgar sense of the word, as commonly understood.§

IV. ——— is as generally curable as any of those violent diseases most successfully treated by medicine.

"§ Mr. Haslam, in attempting to form a table 'wherein might

The first chapter is inscribed, *Insanity has always corporeal disease for its foundation.* Though this may seem the ground-work of all the author's physiological and pathological reasonings, we shall dismiss it very shortly. First, because the chapter abounds with metaphysical reasonings from all sources, as well as from the author himself; and, next, because, in our opinion, the question is of little importance. If madness arises from an organic lesion in any part of the body, it can only be cured by the relief of that organ. If from some impression on the mind, it is of very little importance to consider whether the mind is corporeal or immaterial. Our remedies must be what are usually termed *moral;* that is, they must be directed to the passions in the best manner that we can find of inducing a just impression on the intellect. It cannot be questioned that the body will often suffer from impressions on the senses, or on the intellectual part, and must be attended to at the same time. On this subject we conceive more information is derived from the cases in the Appendix, than from our author's reasoning; but, as he has chosen to place those cases at the conclusion of his volume, we shall defer our remarks till we arrive at that part.

The second chapter is to prove that *Insanity consists of one species under two forms, viz. sthenic and asthenic, or mania and melancholia.* This is divided into ten sections—1st. That a disease under the same name may assume the sthenic as well as the asthenic form: a sthenic disease also passes very often into an asthenic. 2d. Contains the history of the sthenic or high form of insanity. 3d. On the termination of sthenic insanity. 4th. The history of asthenic or low form of insanity. 5th. The termination of the asthenic insanity. 6th. The predisposing causes of insanity. 7th. The proximate causes of insanity. 8th. The exciting causes of insanity. 9th. The lucid intervals, and partial insanity. 10th. The prognosis.

---

be seen the probable direct course of this disease, and also its collateral bearings,' tells us, 'difficulties have arisen.' Truly they will ever arise, for such a table neither can or ought to be formed, for the very reason he has given, viz. 'It appeared, on consideration, improper to attempt precision with that which was variable and as yet unsettled;' and, happily for mankind, will ever so remain. The few instances which Mr. H. with all his undoubted industry, zeal, and experience, in the most extensive field of observation, has been able to select, amount to nothing in proof of necessary hereditary insanity. The instance of M. M. was a case connected with menstruation: after the final secession of this evacuation she recovered, although she had been confined sixteen years. Haslam on Insanity, 2d edit."

The

The first section has for its superscription a text from Struve's Asthenology. This, as we observed before, is only a specimen of the author's fondness for authorities. A few paragraphs at the commencement of the section will further illustrate this, and, perhaps, explain the author's meaning.

"Two families of disease comprehend the whole, says a young ingenious writer, in the Medical and Physical Journal; and he has said rightly. Whatever reluctance may exist in the minds of some medical practitioners to allow the justness, truth, and precision of this simple but solid fact, the foundation upon which the statement rests will bear the most accurate examination, by which test alone can the results to which its adoption leads be justly appreciated. Objections may doubtless be made to any term or terms, however appropriate; but objectors have rarely been fortunate in the art of medicine, when attempting to establish one perfectly conclusive —witness all the futile endeavours to produce a perfect definition of insanity.

"'In diseases the excitement is seldom merely strengthened or merely weakened in the whole system, and the division into two principal forms is by consequence a wrong one.' *Psaff on Brown.* This is certainly attempting to draw an important inference from very feeble premises: it is sufficient that no general disease exists, while equable excitement exists; but excess or defect having once taken place, disease is now present, of what force or kind being determined by other causes or changes not operating against the justness of the arrangement, general ideas, general terms, and correspondent notions must be entertained. The inequality of the excitement in various diseases ought not to nullify this useful leading and clear distinction. It must, however, be allowed, that the opposite diathesis comprehend some distinct branches, to which Dr. Brown paid no attention, as the peculiar states of the absorbent and nervous systems exemplify; but where is or ever was the architect who at once raised a perfect edifice? Is the Newtonian system a *ne plus ultra?*

"Insanity affords no exception to this arrangement: every lunatic belongs to the high or low form, although it often happens that through mismanagement or some secret adventitious cause, a change takes place from one to the other. By the terms sthenic and asthenic nothing more is meant than the opposite states of the excitability of the system at large, at the period of insane attack; or, in the language of Dr. Battie, 'the morbid effects of anxiety (asthenic diathesis), or the preternatural excess of sensation' (sthenic diathesis). 'Another effect of anxiety is the nervous disorder directly contrary to it, viz. insensibility; that is, a preternatural defect, or total loss of sensation' (asthenic diathesis). *Treatise on Madness*, 1758, p. 58.

"Mr. Meynel, the celebrated fox-hunter, amongst some other useful remarks on canine madness, observes, 'There are two kinds of madness, both of which he has known to originate from the bite of the *same* dog' (doubtless the difference of effect was determined by the state of predisposition of the subjects when bitten). 'Among huntsmen, one is known by the name of *raging*' (or the high form); 'the other by that of *dumb* madness' (or the low form). 'In the latter, the nether jaw drops, and is fixed; the tongue hangs out of the mouth, and slaver drops from it: in the former, the mouth is shut, except when the dog snaps or howls, and no moisture drops from it.' *Manchester Memoirs*, vol. 4.

"Many writers have advanced thus far—'Mania is a state of body opposed to melancholia.' Such writers, upon being questioned, would readily allow mania to be a sthenic disease; what then must be its opposite? Truly, asthenic, or, as the experienced author just quoted observes, 'Sensation *too greatly excited* by real objects and its contrary insensibility; or sensation *not sufficiently excited* by real objects, though acting with their usual force. *Battie*, p. 33. Under one or other of these general forms every insane case ranges, nor is any other distinction useful or necessary; on the contrary, the almost endless shades of difference branched out into species by learned nosologists and metaphysicians have no one beneficial tendency, being only calculated to encumber science, disguise truth, render rugged and disheartening the paths of enquiry to young minds, and perplex all just propriety of distinction; affording a continued source of impediments to the acquirement of a better understanding of the disease called mental derangement, or that state of the human frame labouring under sthenic or asthenic disease, accompanied with a greater or less degree of aberration of the mental faculties. Vide the writings of Sauvages, Linnæus, Vogel, and Segar; or those of our countrymen, Cullen, Darwin, and Arnold. Some of these authors had so little mercy on the retentive faculties of their readers, as to give them no less than forty-five species of what they termed 'Maladies Morales;' but to Dr. Arnold it is sufficient to refer the reader, with the reflection of Locke before him—'He that shall consider after so much stir about genus and species, and such a deal of talk of specific differences how few words we have yet settled definitions of, may, with reason, imagine that those forms which there hath been so much noise about are only chimeras.' *Hum. Und.* B. 3. ch. 6. p. 389.

"'Πρόσθε λέων, ὄπιθεν δὲ δράκων, μέσση δὲ χίμαιρα.' 'Philosophers,' says old Burton, 'make eight degrees of heat and eight degrees of cold; but we might make eighty-eight degrees of melancholy.' 'It is of little consequence what physicians say of distinct species of diseases in their mootings and speculations.' *Anat. of Melan. Intro.* p. 9, 29. 'The arbitrary distributions of Sauvages and Cullen were better calculated to impress the conviction of their insufficiency, than to simplify my labour.' *Pinel on Insan.* p. 2. *Davis' Trans.*"

We shall offer also the two following paragraphs, as they seem to compress the author's meaning into a shorter compass.

"Sthenic diathesis, when connected with insanity, corresponds with a state of too great mobility of brainular action, or the 'strong nervous excitement' of M. Pinel, and of the whole nervous system. Hence, while under this state of action, persons are not exposed to the consequences, although they may be to the influence, of the depressing passions: they are not only proof against such influence, but are carried incessantly onward, in despight of their most vigorous attack. 'Raving and violence form its characteristics, and it is usually attended with some degree of fever, and often with a good deal of *bodily disorder.*' *Arnold on Insanity*, *Pref.* p. 20.

"Thus is given a very fair description of the sthenic form of insanity, all ridiculous nosological distinctions apart. At page 23, the asthenic or opposite form is nearly as accurately depicted. Speaking of ideal insanity, he says, 'It is always attended with symptoms of a disordered state of the brain and nervous system, and frequently with disorder in the stomach and hypochondriac region.' *See Dr. Hull on the Nerv. System. Case by Mr. Home, Philos. Trans.* 1801. Hence it will be found that the asthenic diathesis conversely corresponds with a state of too great immobility; the sufferer is not to be raised by the joyous, exhilarating, or pleasurable passions, but he frequently becomes concentrated to a state which has been termed '*stupor vigilans.*'"

Some remarks follow on predisposition, in which the name of Haslam, Darwin, and Arnold, all occur in a single paragraph; but, as this appears to us an anticipation of a succeeding section, we shall dismiss it till it occurs in order.

We shall quote a short passage from the next section, on the terminations of sthenic insanity, and compare it with another in the foregoing section, before we offer a remark, to which we sincerely wish the author had been more attentive.

"Mania, or the sthenic form of insanity, very frequently terminates in the opposite form, or melancholia. Chronic incurable derangement, or fatuity, embraces a large proportion of its subjects. In paralytic affections as in Case, No. 16. *Append.* A few cases in sudden death, as from a stroke of apoplexy, Case No. 11. *Append.* The remainder of a given number are restored to health. Original neglect or bad treatment may produce or accelerate all the unfortunate terminations; sudden inanition destroyed numbers of wretched mortals at the *Asylum de Bicetrè.* In the year 1784, 57 died out of 110—1788, 95 out of 151; but when better fed, one in eight only was the annual proportion. *Pinel*, p. 32.

"Sthenic insanity is very readily and frequently converted into the asthenic, by great and sudden prostration of strength, the conjunct effect of various violent evacuations: the same mode of [illegible]actice persisted in for a considerable length of time in a vigorous young young subject, or for a shorter period in an older and less powerful one, will produce fatuity or paralytic affection. M. Pinel, with his accustomed sagacity, cautions practitioners against inattention to the dangerous debility which so often supervenes on the decline of mania; and justly observes, 'it is a state requiring peculiar attention,' to prevent, at this critical juncture, the increase of the melancholy list of idiotic and chronic lunatics."

"Chronic maniacs, originally seized under circumstances strictly sthenic, will endure, without noticing it, what would be a very inconvenient degree of heat or cold to a sane person."

Should another edition be called for, we submit it to the author whether it would not be better to make his division of madness into acute and chronic, and afterwards sub-divide each, particularly the latter, into its various forms. We admit, with him, that many of these sub-divisions have been worse than useless, and that the terms are often irksome, if not ridiculous; but where a practical difference occurs in the prognosis or mode of treatment, no one can question the importance of distinctly marking every distinction which may be traced with any certainty. We must not, however, pass over one symptom, which Mr. Hill remarks in many of his cases; but which we do not recollect to have been generally noticed.

"The cutaneous glands and *materia perspirabilis* in this [the sthenic] form of insanity, become greatly altered, sometimes early in the disease, although not much noticed at any period: hence this disorder, like others of a severe nature, is found to have its peculiar attached odour (*'olere hircum'*) arising from the several emunctories, and is more strikingly obvious to the olfactory sense of the observer under this form than the opposite. *Bacon*, v. 1. *Cent* 3. p. 198."

In many acute diseases, the *perspiratio olida* has been attended to by the best physicians, and usually as a favourable symptom: we think our author not sufficiently decided in this respect. We refer to his chapter on prognosis.

Mr. Hill considers the usual termination of asthenic insanity to be "apoplexy, palsy, epilepsy, dropsy, phthisis, fatuity, chronic lunacy, and suicidical death, rarely in health, unassisted by art, or in what may be termed natural sudden death." We cannot help thinking that all this might have been said of chronic insanity, instead of making it one of the terminations. We are the more confirmed in this opinion by the concluding sentence of the section, viz. "Modern annals testify that lunatics, generally speaking, inherit a protracted existence. This can only be said of the chronic form of the disease."

The sixth section, on the predisposing cause of insanity apr[illegible]

appears to us very much confused. The term predisposition, we are told, is understood to imply a state short of that state to which it tends, and incapable of giving that which it has not itself attained. This is illustrated by a case, which we shall transcribe.

"CASE XXVI.—Miss F., æt. 23, of a lively pleasant temper, generally healthy up to this period, of a robust form, received a severe blow on the back part of her head, as she was rising up suddenly, and had forgot a piece of timber above. She was not stunned at all, nor did she complain for several days. The frequent application of the hand to the stricken spot first excited notice; subsequently an alteration in her behaviour gradually followed. Accidents, or new circumstances of any kind, greatly affected her. Thus arose much inequality of temper, stupidity, inertness, and close silence. Occasionally, for a very short time, a volatile flightiness. The opposite situation was accompanied with a peculiar feeling in the head, wholly unknown before the blow. When this receded, which it would sometimes do instantaneously, at others slowly, her intellects seemed nearly, but never completely perfect. Twelve months subsequent to the accident, I first saw her, and took much pains with the case unsuccessfully. All her conduct seemed to depend upon the ebbs and flows of the peculiar feeling. Yet this conduct, however distressing, could not strictly be called madness, though the mind aberrated occasionally, from the result of local mischief, varying with all the varying states of the excitability independant of insane predisposition, yet illustrating what must have been the consequences had such predisposition existed at the time the blow was received. The lady remains precisely as she did in 1797."

Whether this conduct could be called madness, we shall not take upon ourselves to determine; but we know not why it should be called predisposition to that malady. *Pre*disposition seems to us something which *pre*cedes disposition, and disposition that state in which a part or the whole is so disposing itself as to take up a certain change. Every human embryo is *pre*disposed to respiration; but the disposition does not take place without certain changes, after which respiration must follow. Every animal is *pre*disposed to the propagation of the species; but the disposition does not exist till certain changes take place; in other words, till the age when certain parts are so *disposed* to take on that act. In disease, this is best illustrated in the contagious. All the human race is *pre*disposed to receive the small-pox, but the disposition does not take place till the subject is exposed to the contagious matter, after which, in a given time, the diseased action follows. Hence it appears to us that all accidental causes should be rather said to excite the disposition in a subject having originally the predisposition. That is, these these causes would produce only a slight, or, at most, only a temporary, effect in those who are not predisposed to high action of any kind, but in others would produce a disease correspondent to their original predisposition. In the next chapter, on the proximate cause of insanity, the author seems to admit all that we require.

"Excessive mobility and immobility (says he) of the brain and nervous system are opposite states, constituting predispositional tendency to actual disease corresponding with excessive and defective excitability, but are insufficient to explain the proximate cause."

If we understand this passage, it must mean that some *original* condition of the brain constitutes the *predispositional* tendency to madness. Mr. Hill continues—

"The common definition of the proximate cause of a disease is 'That which at present forms the disease, which, when changed, changes,—and which, when removed, removes, the disease:' in fine, proximate cause is itself the disease, for, if absent, the disease is likewise absent; predisposition alone precedes disease, and this necessary state, together with the exciting cause, is all we can, in point of fact, be said (and that but partially) to know. Upon considering these facts with all the care they demand, it appears deducible that the *causa proxima* of insanity consists of a *peculiar* or *specific change in the power of accumulation* and *subsequent action of the subtle* 'matter of nervous influence,' of which we know so little, yet write and talk so much, which has been designated by a host of epithets unnecessary to enumerate. *Struve Asthenol.* p. 37."

This specimen may serve to show how little is to be learned from this chapter, which, to do the author credit, is a very short one. To us, however, the proximate cause appears a most important consideration in all diseases: not, perhaps, the proximate cause of the disease, but of the more striking symptoms. Thus, if, after death from phrenitis, we see effusions, extravasations, and adhesions, and know that these are the usual progress of inflammation, we have now learned what are the proximate causes of the symptoms during life, and how to remove them should they occur in any future subject. After our former remarks, it will be unnecessary to say any thing on the section concerning the "exciting causes of madness." The next, on lucid intervals, contains some very conclusive arguments on this important question. And the concluding division of this chapter, on the prognosis, is replete with very useful hints, though much novelty cannot be expected.

The third chapter is on insanity as an hereditary disease. Here our author might have saved much time, had he attended to the distinctions we before marked out, and for which we are indebted to Dr. Adams's "Treatise on the Hereditary Peculiarities of the Human Race." That an uncertain number of individuals of certain families fall into certain diseases from very slight causes, cannot be questioned; but, as the number of individuals is uncertain, as the degree of susceptibility is uncertain, and as the exposure to the causes is not only uncertain, but may, in many cases, be prevented, we agree with our author that it is highly improper to make use of such expressions as hereditary *diseases* or hereditary *taints*. We wish, however, we could have found that perspicuity on so important a question which we expected to follow a reference to our distinguished English physiologist Mr. Hunter. We shall transcribe the paragraph, which, with our remarks, will give the fairest opportunity of illustrating the subject.

"Insanity (says Mr. Hill) may be acquired by accidents of a very violent nature, suddenly applied as exciting causes, acting upon a mild degree of lately acquired predisposition, e. g. The unaccustomed use and abuse of diffusible stimulus, deranged hepatic function, and some unexpected sudden action of one or more of the passions. Now, children born anteriorly to such a misfortune of their parent, (and it is not an uncommon one) cannot inherit insanity by hereditary predisposition. One of the most acute anatomists and able physiologists this country ever produced, said, on a solemn public occasion, 'There is *no* disease acquired which can be given to a child; *there is no such thing as an hereditary disease*, but there is an hereditary disposition for a disease.' *Hunter's Evidence on Donellam's Trial*, p. 51, fol. edit. Therefore all that can be said as to this disposition is, that insanity will be produced the more or less readily from similar causes, operating upon different bodies, in exact proportion as they are similar in resemblance from whatever stock they may have descended, all other causes favourably combining to produce the disease, which similarity does not happen one time in a thousand, but when it apparently does, the doctrine of hereditary disease is revived, but it does not, in fact, ever take place of mere necessity, even in connexions where the ancestry on both sides may have afforded cause of suspicion. It is rationally conclusive, then, that the belief in necessary, absolute, hereditary madness, is a most injurious unphilosophical tenet, and that all deductions founded thereon are hypothetically erected 'upon the supposition of a principle of whose existence there is no proof from experience.' Pure hereditary insanity and pure mental derangement rank equally in the number of gross errors."

Every reader must perceive, if not a contradiction, at least a confusion, in the above passage. It is enough that "these accidents of a violent nature are applied to a mild degree of predisposition, which predisposition must be original, and not not acquired." The diffusible stimuli, to adopt the above language, is an exciting cause, and produces the disposition; for no other cause is necessary to induce the disease, but a continued use of such stimuli. When Mr. Hunter says that no *acquired* disease can be given to a child, he must include madness *acquired* by the free use of stimulating liquors. Whether, therefore, the children are born anterior or posterior to madness acquired in this way, is of no consequence; nor, indeed, is it of the least importance whether children born of parents predisposed to madness are born before or after the predisposition has been excited into action, because the predisposition alone is hereditary, the existence of which is only proved, but not acquired, by the subsequent appearance of the disease.

The length of our remarks, and these copious extracts, render it inconsistent with our limits to dwell on the remaining chapter. It is principally confined to the cure, which may be considered by some as the most important part, but we trust that most of our readers are aware that the nature and history of a disease being known, the mode of treatment is readily understood. To use the words of our English Hippocrates—*Si morbi cujusdam historiam accurate scirem, par malo remedium haud dubitabo invenire.* It is enough to remark, in general, that we have found, in this part of the work, the directions rational, the remarks candid, and the inferences fair. We are particularly pleased with the cases contained in the Appendix. They seem collected with great attention, and narrated with as much brevity as is consistent with perspicuity.

We regret that the work abounds with many errors of the press, which, probably, arose from the distance at which the author was from his printer, and the want of a friend to revise the sheets after his correction. Some of these could never have been overlooked on any other terms. We shall instance only two, in order to show that our remark is not captious. In page 97 we read of "*uncommonly singular* dreams." Page 418, "A widow was left to conduct a large concern, with an only child, which she executed very cleverly: she was of a swarthy complexion, full eyed," &c. Nobody will suppose that she executed her child very cleverly; but many readers may doubt whether the mother or child was of a swarthy complexion, full eyed, &c. These, though trifling errors, very much deface a work which has evidently cost the writer much time and pains, and abounds with considerable information.

*Edinburgh*

*Edinburgh Medical and Surgical Journal*, No. XLVI. for April, 1816.

(Continued from p. 408.)

Art. II.—*Observations on the Utility of Blood-letting and Purgatives in a Fever which prevailed in the Russian Fleet.* By D. J. H. Dickson, M.D. F.L S. Physician to the Fleet, and formerly Superintending Physician of his Imperial Majesty's Squadron in the Medway.

If the paper from this Journal, noticed in our last Number, showed some marks of youthful fervour, the present is not less distinguished by a gravity highly becoming the subject, and suited to the rank of Physician to the Fleet, &c. On the proofs in favour of the practice mentioned in the title we shall say less, because the question is now scarcely disputed; but there is a number of minuter points which we are glad to see in print, and some opinions on the doctrine of contagion, which we have now a happy opportunity of contrasting with the contents of the immediately preceding article.

We copy the following passage to show the unfortunate influence of *slang*, to use a maritime vulgarity, and to avoid a coarser word. The reader will perceive that we refer to the sentence within the inverted commas, which the author has offered as the language of his predecessors.

"When (says Dr. D.) I joined the fleet, the fever, in both instances, had become less typhoid; it was reported to me to be 'synochus, frequently terminating in typhus, and death, if copious evacuations had not been had recourse to at an early period of the disease.'

"It is highly important, here, to contrast the difference of symptoms under this practice, even in unfavourable cases, with those of the patients first received, which had not been controlled by depletion. The tongue was often parched, but not black; there was delirium, but not of the low muttering kind; subsultus tendinum, but without great nervous tremor; seldom involuntary discharges, and no strabismus, nor those appearances of putrescency which mark the close of malignant fever. It appears to me, therefore, an inevitable conclusion, that, by those remedies which repress inflammatory action at the commencement, those graver, or eminently typhoid symptoms, which characterize the advanced stage of such fevers, were prevented. But I am not anxious to designate the disorder, since it has been but too common to connect with the name peculiar ideas of a disease, modifying the treatment, which ought alone to be regulated by a knowledge of its nature and tendency, aided by the pathological light of dissection.

"In proof of the highly infectious nature of this fever, in the first instance, particularly in the hospital-ships, where so much disease was concentrated, it will be sufficient to mention, that nine out of eleven medical officers, attached to the sickly division, and to the hospitals, were attacked in the course of a few weeks. It proved fatal to one surgeon and an assistant; and two assistant-surgeons belonging to the Trusty died of the consequences. I was one of the last taken ill, after having been exposed for little more than ten days; which may be readily accounted for, when the powerful exciting causes inseparable from visiting a distant, detached, and sickly squadron, in the middle of winter, are taken into consideration.

"The contagion appears to have been particularly powerful in the Trusty, which ship had received the first, and consequently the worst cases; as not only the surgeon, and other medical officers, suffered severely, but twelve out of sixteen attendants, accustomed to the duty of waiting upon the sick, were seized with fever, four of whom died.

"Several attendants in the Argonaut, two assistants, and ultimately the surgeon, were also attacked; but he informs me, that, with one exception, all recovered, in whom he had the advantage of combating the disease at its commencement. It is needless here to dwell upon the value of various prophylactic measures under the heads of separation, ventilation, dryness, cleanliness, better clothing, &c. to which my solicitude was chiefly directed, or the difficulties that opposed their execution. I may, however, remark, that the benefit was in proportion as they were practised, and that the disease gradually became milder, to which the decreasing severity of the weather, as the spring advanced, materially contributed. Indeed, although malignant cases still continued from time to time to occur, in the more general course of symptoms, such as I am about to describe, there was little remarkable; and, without keeping in view the tendency to inflammation and disorganization which characterized the progress of the disease, there would have appeared little to warrant apprehension; but, as Dr. Haygarth justly remarks, after describing the symptoms of Typhus mitior, sometimes even this mild typhus is fatal."

After what we have said of *typhus*, we shall not stop to inquire how a disease can be at once *mitior* and *lethalis;* but confine ourselves to a few remarks on the contagious property of the fever. The writer of the *preceding* article remarks, that, in the fever of which he had the charge, "not a single individual of those who had hourly intercourse with the patients, was ever seized with the disease. If even it had been otherwise," he adds, "I am far from being disposed to grant that contagion was thereby proved, for where all are equally exposed to the peculiar causes, seizure must often be indiscriminate." May not the same argument be urged against Dr. Dickson's proof? But we have another, and, in our estimation, still greater objection to the indiscriminate use of of the terms infection and contagion, contained in the extract from his paper. In a hospital ship, crowded with sick, during the winter season, must we not expect a foul air, and that such a cause, when generated, would be equally dangerous to all who approached within the sphere of its influence? We might also conclude, not only that the danger to the attendants should be less, but even that the subsequent fever would prove milder in proportion as ventilation was more attended to, and as the warmth of the season permitted the adoption of such means. But is this the case with the contagions strictly so called? Does ventilation lessen the contagious property of small-pox? or is the disease rendered milder in proportion as the effluvia inhaled are more diffused in common atmosphere? On the contrary, do we not find that the disease, taken in the casual way, at such a distance from the source as induces a dangerous security, is often as fatal as when received at the patient's bed-side. This is all we shall offer at present, because we have promised a distinct dissertation on the subject of contagion, after we have noticed several other performances on this important question.

We shall now, with much greater satisfaction, turn the attention of the reader to some valuable practical remarks of this ingenious writer. Though the necessity of bleeding and purging is now pretty universally admitted, in many fevers, from whatever cause, yet the manner of conducting it in the different stages is no where better explained than in the present work.

"In the Trusty, the treatment deemed the most successful was, where the patient lost, within the first week or ten days, between sixty and eighty ounces of blood, by taking away from sixteen to twenty-four ounces at a time. When the fever was violent, a larger quantity was abstracted in a shorter period; but it was not often necessary to exceed thirty-six ounces in twenty-four hours, at two or three bleedings. After the tenth day it was not often considered proper to bleed, or only in smaller quantities, when indicated by symptoms of pressure upon any particular organ, or by the appearance or renewal of inflammation. When this remedy had been neglected at the beginning, or the patient was admitted on an uncertain day of the distemper, small bleedings of six or eight ounces, repeated according to the effect, were found safer than larger ones, which might have proved too debilitating, and were serviceable in preventing or moderating the consequences of inflammation and congestion. An able physician, Dr. Parry, in his Elements of Pathology, page 317, thinks 'it is probable that subsultus tendinum, convulsive motions of the limbs, and hiccup, which often concur with delirium in various fevers, arise from long or violent irritation of the brain by sanguineous impulse.' It is certainly certainly in favour of this idea that effusions of blood or serum were generally found in the brain of those who died with these symptoms about the 18th day; and, that, in some patients, where small bleedings, graduated by the pulse, were tried even as late as this, when delirium, subsultus, startings, and coma, indicated an oppressed or irritated state of the sensorium, these symptoms were diminished, the respiration became freer, and the intellect more distinct after its employment. Under this treatment, some apparently hopeless cases assuredly recovered; but it oftener failed. It is allowed that the presence of fever is most certainly detected by the state of the animal functions, and of the pulse; but to the latter there are many exceptions. I need not here adduce the many authorities that might be quoted in support of my own observation, to shew that the pulse, in many cases, has been found little affected in the worst fevers; that it is often little, if any, quicker than natural; and that it is sometimes preternaturally slow. But I believe it to be unnecessary to dwell upon its fallaciousness, or on the little information it often affords as to the propriety or quantity of blood to be taken, particularly where the head is much affected. In the early stage of disease it is often small, low, feeble, and irregular, previous to considerable re-action; but, when the accession of this state is characterized by increased heat, hard, full, and frequent pulse, throbbing of the carotids, and other symptoms of excessive determination, the indication is sufficiently manifest. This state of increased action, however, does not always follow, but the pulse continues low and contracted, or labouring and oppressed, until relieved by evacuations, when it rises, becomes fuller, and more equal;—an effect which, with correspondent improvement in the intellectual powers, I have often seen produced by purgatives, as well as by venesection, in tropical fevers.

"In this depressed state, the employment of a remedy, by no means passive, requires nice discrimination; for it is necessary to distinguish between that period of diminished energy preceding re-action, where it would prove injurious, and that in which, to use the language of Sydenham, 'all the symptoms of weakness proceed from nature's being in a manner oppressed, and overcome by the first attack of the disease, so as not to be able to raise regular symptoms adequate to the violence of the fever,' until 'it could disengage and shew itself' by bleeding. Vol. II. p. 351.

"It is impossible, therefore, from the state of the circulation, to lay down any infallible criterion for the employment of blood-letting in fever. The safest is the hardness of the pulse, and a white tongue, as indicating inflammatory action; and, upon the whole, it was generally considered at least safe to bleed in the early stage, where the heat was increased, and the pulse above 100.

"The degree of resistance of the artery against the finger was considered a better guide than the size of the pulse; if it was firm and equal, bleeding was generally proper; if easily compressed, soft, or undulating, the contrary; if it felt tense, or corded, or the the stroke was described as sharp, harsh, jerking, or rebounding, it was considered indispensable; but, in using such terms, we must be aware how difficult it is to attach precise and determinate meanings to words, and that the same pulse will be described very differently by different reporters.

"In speaking of the fallacy of the pulse, I ought not to omit noticing the unequal distribution and power of the circulation which not unfrequently obtain in fever, as another source of error, if we judge of its force, in the vessels near the heart, by those of the extremities; for it may be strong and bounding in the central, yet weak and languid in the distant arteries. Some marked examples of this kind occurred from exposure to severe cold for several hours in boats after depletion; in consequence of which I grounded my application of the necessity of having a decked vessel to convey the sick to the hospital-ships. These patients, notwithstanding the application of warm blankets, &c. continued to complain of an extreme sense of chilliness, with coldness, and a sunk languid pulse in the extremities, while the face was hot and flushed, and the large vessels of the neck and head were greatly excited, indicating what Mr. Hunter calls action without power, and showing the danger, in such a case, of appreciating the state of the internal circulation by that of the radial artery. This unequal and partial distribution of heat, which seems to have engaged the attention of the ancients much more than the pulse, is very unfavourable in fever; and the same is the case whenever the actual condition of the patient and his feelings are much at variance; as, for example, when he complains of a much greater degree of either heat or cold than is indicated by the touch or by the thermometer.

"With respect to the comparative advantages of large or of frequently-repeated small bleedings, in early fever, both plans were employed here, and with various results; the latter may be often useful and safe, where the former would be inadmissible. But, at the commencement of the attack, or where some important viscus is threatened with inflammation, I must give a decided preference to the large and sudden abstraction of blood, while there is yet any chance of anticipating or removing congestion, or of cutting short the fever. The one will, of course, be preferable while we have these objects in view; the other may be useful in mitigating symptoms where the expectation of crushing the disease can be no longer indulged. It is also evident, that the occurrence or renewal of inflammation later in fever may justify a cautious and limited detraction of blood, when the loss of a larger quantity could not be borne."

Some very useful remarks follow on the different appearance of the blood in different stages of the fever. In the beginning it often exhibited only a healthy appearance; in the more advanced stage, in the same subject, it exhibited every mark of inflammation. This difference has been accurately noticed by the great physiologist above referred to.

4 In

In the beginning of local inflammation, Mr. Hunter remarks, that blood will show no marks of increased action; in a few hours after, we shall find blood taken from the same vein buffy and cupped. This difference he imputes to the first blood that was drawn not having yet sympathized with the inflammatory action which has affected a solid part. In this manner we are often deceived. The parenchematous part of the liver or spleen has very little sensibility; until, therefore, the whole system sympathizes, few indications of inflammation are discoverable; and, in a more advanced state, delirium will sometimes, to an unexperienced practitioner, confound all the symptoms. Hence the danger of the slightest delay in the earliest stage of fever.

The author's remarks on petechiæ are not less judicious. In the early stage, they mark often increased action, and are removed by free depletion. In the more advanced stage, they threaten more danger. We must, however, add, that their colour ought always to be attended to. When purple at any period, they must be considered dangerous, when florid, less so, or not at all, and the various shades between may be said to mark the degrees of danger.

With these well-founded remarks, many of which, though often occurring, are overlooked by more hasty and superficial observers; we were concerned to see that the author should not have courage to discard expressions which he must have lived long enough to adopt or not, as he pleases.

"In a *typhus* fever (says he) which afterwards prevailed among the Danish and American prisoners of war, and in which a glossy and turgid appearance of the eye was often the first indication of the disease, Dr. Dobson, of the Trusty, informed me, that he found venesection attended with the same success as in the Russians; but, while he is firmly of belief that no other plan was equally successful, he candidly acknowledges, that, in many cases, his expectations from the lancet were altogether disappointed, while, in others again, it seemed to save several who were studded with petechiæ,—a symptom that often manifested itself within thirty-six hours of the attack. After reverting to the failure of the lancet in cases where he had reason to expect success, he concludes,—'But these failures by no means argue against the propriety of the practice, where no other measure was equally successful; inflammation was still present, and to its consequences death was, in every case, clearly proved by dissection.'"

Some ingenious enquiries follow on the connection between fever and inflammation, these we conceive more the offspring of modesty than decision. That fever is always attended, in some part, with increased action where it is a curable disease, can scarcely be doubted, or that in all such cases the principal danger is, lest that action should be carried too high in some some particular organ. On the other hand, fever, which is no more than universally altered action, may be attended with extreme debility, and the cause itself may be sufficient at once to render the whole system incapable of those functions by which life is supported. In these cases, we find a rapid extinction of life preceded by a change in the properties of blood, which renders it incapable of oxygenation.

What we have here suggested, must rather be considered as hints than systematic reasoning; and, if we are disposed to differ with Dr. Dickson, it is only because, with extensive practical knowledge, we wish him to assume a language suited to the clearness of his conceptions, the minute accuracy of his observations, and the general perspicuity of his reasoning.

Art. III.—*On the Malignant Fever which prevailed at Gibraltar in* 1813, *and its Treatment by Blood-letting*; by J. Humphreys, Assistant-Surgeon, Royal Artillery.

The remarks on the treatment of this fever are less important in proportion as the subject is become better understood. We cannot, however, have too many well-grounded confirmations of a fact, the application of which has been so calamitously slow in the army practice. But the question of contagion renders the present paper peculiarly interesting.

"On the first appearance of the fever in 1813, (says Mr. Humphreys) in its aggravated type, a report was spread, and generally believed, that it was contagious; the garrison was immediately placed in a state of the strictest quarantine, and families became so exceedingly alarmed from a supposition of its being highly so, as to shut themselves up, refusing admittance even to their friends. I do not, however, concur in this opinion, and hope to show, in a satisfactory manner, by many instances which I shall adduce, that no contagion existed. The disease, at its commencement, attacked persons in different parts of the town at the same time, who were very remotely situated from each other, and those who shut themselves up, and considered themselves secure, being perfectly instulated, were attacked as readily as those who mixed indiscriminately with the people as usual; shewing clearly that the disease was produced by general local causes, acting on the whole population of the place, and not imported, of which no satisfactory assignable source has ever been traced; nor do I think it probable that contagion is of that peculiar nature, as to show itself in different years precisely at the same season, having lain dormant and inert nine months of the year, and then burst forth to commence its devastating influence.

"The local causes which appear to me to have given rise to this so much dreaded and formidable disease, are principally the following:—The excessively crowded population of the place; the houses and sheds being literally huddled together in so limited a spot, without arrangement, and of the worst construction; the doors and windows being generally on the same side of the apartments, so as to preclude a free access of air and ventilation; and among the lower orders of Portuguese, Spaniards, and Jews, these dwellings are usually shut up during the whole of the day, from sunrise to sunset; consequently, on their return, they must breathe a very impure air, almost wholly deprived of its oxygen; the immense collection of animal and vegetable matters, arising from so great a population, during the dry summer months, remaining stagnant from May till the end of August, and which become roused into action at the end of the autumnal season by the partial rains, when the quantity of water is not sufficient to propel them through their respective drains, which are too narrow for their evacuation, being frequently choked and bursting. At this season, also, the heavy night-fogs, succeeded by a parching sun, occasion exhalations extremely noxious and unwholesome. To these may be added the peculiar situation of the town, which is on the western foot of a steep rock, about 1400 feet in height, running nearly north and south, the air remaining nearly stagnant during the prevalence of the easterly winds, which continue with but little variation the whole summer.

" Having enumerated such of the local causes as I conceive to be the leading and most prominent, in aggravating the bilious remittent fever, which we here witnessed in the years 1813 and 1814, I shall now proceed to state some striking examples.

" A lady, an officer's wife, residing in the Moorish Castle, which is considerably above and out of the town, never left the castle, and was so alarmed that she would not allow any individual to approach her; and, after the fever had existed five weeks or more, she fell ill and died of it; her husband, who was constantly at her bed side during her illness, escaped the disease.

" Similar precaution had been taken by the Ordnance storekeeper, Robert Pringle, Esq. who had adopted the most rigid quarantine for more than three weeks, yet he did not escape. As his was the first case where I ventured to deviate from the common mode of treating the disease, and as it terminated successfully, contrary to the opinions of the medical officers, and others who knew him, I take the liberty of giving an outline of it. He was seized suddenly with the common symptoms, as cold shiverings, violent pain of the head, loins, and calves of the legs; great pain and redness of the eyes, dimness of sight, vomiting, hot dry skin, coated tongue, and quick strong pulse. I took away immediately forty ounces of blood. After the loss of twenty ounces, he expressed great relief, and begged of me to continue it, which I did till the above quantity was abstracted. His head became instantly relieved; he could see distinctly, and his pulse, from 125, was reduced to 90. I ordered him a strong purgative of calomel and jalap, and cold water, with vinegar, to be applied to his forehead. Four hours afterwards I visited him again. Found that his head was was entirely relieved, but some pains of the loins and calves of the legs remaining. He had had some sleep, his skin was moist, his bowels were moved, and his pulse nearly natural. He was directed to take three grains of calomel every three or four hours, and on the fifth day he was perfectly recovered. Mrs. Pringle, who was constantly with him, escaped, as did the children."

Many similar instances are produced.

Art. IV.—*Proofs of the Bulam Fever attacking the Human Frame only once;* by W. Pym, M. D. Inspector of Hospitals.

This is a very important paper. The facts brought forward in proof of the premises are numerous, and, we cannot doubt, not less correct. We shall offer the two concluding paragraphs, which contain the author's claim, not to the discovery, but, which is still more important, to its practical use.

"I am truly surprised that its peculiarity of attacking the human frame but once, has not been sooner known; and now, that it is mentioned, that it has not excited greater attention. Lining mentions it particularly, in the Edinburgh Medical Essays, fifty years ago. In Sauvages' last editions, about 1768, it is positively mentioned. The English have long known the fact under the name of *seasoning*, and the French of *tribut, acclimaté,* or *une idiosyncrasie réfractaire à la contagion.* Monsieur Berthe, in mentioning the disease at Cadiz, says, "Le petit nombre de ces individus *ainsi priviléglés* a été observé parmi ceux qui avaient habité les Antilles.' The emigrants from St. Domingo were proof against the contagion of Philadelphia in 1793-4. But vaccination was long known in Gloucestershire to the dairy-men before Dr. Jenner's discovery; and, with respect to my discovery in the yellow-fever, I cannot give it up to the Spanish practitioners. I made the discovery that the West Indians were proof against it on the 20th October 1804, or rather the 19th, for that was the day that I requested Sir Thomas Trigge (governor) to order the men who had been in the West Indies to be paraded.

"The first Spanish physician that mentioned it was Arejula, and he did not publish until 1806. Sir J. Fellowes gives the credit of it to the Spanish physicians generally. No individual one has claimed it. It certainly was not known among them in 1803; nor do I believe it was ascertained in 1804, until after the time that I discovered the non-liability of the West Indians, when I requested my friends in Gibraltar to write to Malaga and Cadiz, where inquiry was made, and the fact proved."

*Rudiments of the Anatomy and Physiology of the Human Body, consisting of Tables, &c. compiled for Students of those Sciences, beginning their Researches.* By T. J. Armiger, Member of the Royal College of Surgeons, Surgeon Extraordinary to the Duke of Kent, &c. &c. late Demonstrator of Anatomy at the London Hospital. Cox and Son, 1816; pp. 52.

This is a most valuable little compilation for either the *emeritus* or the student. For the former, if not constantly engaged in operating or dissecting, nothing can be more convenient than so general and so convenient a reference to parts by their name. For the latter, the advantages are still greater. Before lecture, he may be prepared for the subject, and after lecture, by reviewing, he may avoid the custom of making notes, which always endangers the attention, but particularly when there is a demonstration. All this is well expressed by the author.

"These rudiments of the anatomy and physiology of the human body, are intended for students of medicine beginning their inquiries in those sciences. They have been compiled to supply a deficiency of our elementary books, which do not proceed progressively from explained to unexplained truths. 'The student will ever make the most progress, who, rising from less to greater points, and from the more easy to the more difficult, moves on in regular and happy gradation.' It becomes me to acknowledge that the materials are collected chiefly from approved authors, and to state that my readers are to expect no other novelty than the method of arrangement."

A short introductory chapter follows, containing all that is necessary previously to entering on the subject. Next follows a general division of the body—its component parts—character of the various textures—of its functions—of the organs by which such functions are accomplished. These are reduced into a compressed and perspicuous table, succeeded by another on the distribution of these organs, according to their various situations in the body.

A description of the bones follows, which is preceded by a very proper caution, lest the student should confound the artificial with the natural skeleton. This part of the work concludes with an account of the connection of the bones, in which the author has, of course, introduced all the technical terms by which the joints are distinguished, as well as their component parts, and fluid contents.

A fifth table contains the muscles of the skeleton. This method of arrangement is adopted to avoid the use of such terms as, origin and insertion. The tables contain, on one side, side, the name of the bone in which the muscle is usually said to have its origin; on the other, its insertion; and in the centre, the name of the muscle itself. This is not only the most simple arrangement, but the most perspicuous, and, beyond comparison, the most useful, as it introduces the reader at once to the situation and uses of each muscle.—Tables of the arteries, and of the veins, are not less judiciously arranged. A view of the absorbent system follows; and the whole concludes with a similarly constructed table of the nerves.

Of the merits of this work, the most striking are brevity, perspicuity, and judicious arrangement, which are, indeed, all that the author promised. We think no student should engage in a course of lectures, or dissections, without supplying himself with it; and doubt not that the encouragement with which it is received will induce the author to continue his labours.

